# Bedtime Audiobook Listening Motives and Insomnia Status: Survey Study

**DOI:** 10.2196/95064

**Published:** 2026-08-10

**Authors:** Karl Berglund, Elisa M S Meth, Pei Xue, Christian Benedict

**Affiliations:** 1 Department of Literature and Rhetoric, Uppsala University, Box 632Uppsala, SE-751 26, Sweden; 2 Department of Pharmaceutical Biosciences, Uppsala University, Husargatan 3, Box 591, Uppsala, 751 24, Sweden, +46 70 425 0215

**Keywords:** audiobooks, sleep, sleep-onset insomnia, bedtime routines, adults, Sweden

## Abstract

**Background:**

Many individuals use audiobooks at bedtime to unwind and facilitate sleep, yet little is known about how this practice relates to sleep health. Understanding these patterns may inform behavioral strategies to support sleep.

**Objective:**

This study aimed to examine associations between bedtime audiobook listening, perceived sleep benefits, and sleep-onset insomnia symptoms in a large adult sample.

**Methods:**

A cross-sectional online survey was conducted among 1136 Swedish adults (aged ≥50 years: n=600, 53%; women: n=1027, 90%). Recruitment took place in Sweden between January and February 2026 through social media, university campus flyers, and book-related media outlets. Respondents reported bedtime audiobook listening frequency, motives, preferred genres, and sleep characteristics. Sleep-related variables included the frequency and duration of difficulty initiating sleep. Sleep-onset insomnia symptom status (absent, subthreshold, and clinical level) was derived using symptom-based criteria. Clinical-level sleep-onset insomnia symptoms were defined as difficulty falling asleep ≥3 times per week for ≥3 months, and subthreshold sleep-onset insomnia symptoms were defined as either difficulty falling asleep 1 to 2 times per week for ≥3 months or ≥3 times per week for <3 months. Modified Poisson regression with robust variance estimation was used to examine associations between audiobook use, perceived sleep benefits, and insomnia symptom status, with adjusted prevalence ratios (aPRs) and 95% CIs reported.

**Results:**

Among the self-selected sample of 1136 Swedish adults, 53% (n=601) listened to audiobooks daily at bedtime. Sleep-onset insomnia symptoms were common in this sample (those reporting clinical-level or subthreshold symptoms: n=427, 38%), although these estimates should be interpreted in the context of the recruitment approach and may not reflect population prevalence. Daily listeners had a higher prevalence of reporting that audiobooks helped them fall asleep (aPR 1.32, 95% CI 1.21‐1.44) and improved overall sleep (aPR 1.73, 95% CI 1.41‐2.12), whereas clinical-level sleep-onset insomnia symptoms were associated with a lower prevalence of reporting perceived benefit for falling asleep (aPR 0.93, 95% CI 0.87‐0.98). Daily listeners also had a higher prevalence of reporting difficulty falling asleep without an audiobook (aPR 1.93, 95% CI 1.67‐2.22). Participants with sleep-onset insomnia symptoms had a higher prevalence of using audiobooks to reduce worry or racing thoughts (subthreshold symptoms: aPR 1.34, 95% CI 1.16‐1.55; clinical-level symptoms: aPR 1.28, 95% CI 1.10‐1.49), while daily listeners had a higher prevalence of selecting the same audiobook at bedtime (aPR 1.13, 95% CI 1.00‐1.26) and a lower prevalence of preferring nonfiction (aPR 0.61, 95% CI 0.49‐0.77).

**Conclusions:**

Bedtime audiobooks may be perceived to serve different roles depending on sleep health, with cognitive regulation motives more frequently reported among individuals with sleep-onset insomnia symptoms. Owing to the cross-sectional design, it remains unclear whether audiobook use influences sleep-related experiences or whether individuals with particular sleep characteristics are more likely to adopt audiobooks as a bedtime strategy.

## Introduction

Insomnia is one of the most prevalent sleep disorders worldwide. Global estimates suggest that approximately 16.2% of adults meet criteria for insomnia disorder [1], while a substantially larger proportion report insomnia symptoms that do not reach the full diagnostic threshold (subthreshold insomnia) [2]. Difficulties initiating sleep are among the most commonly reported complaints and are frequently linked to heightened presleep cognitive arousal, including worry and racing thoughts [3].

According to international guidelines [4], the first-line treatment for insomnia is cognitive behavioral therapy for insomnia (CBT-I). CBT-I typically consists of psychoeducation, sleep hygiene recommendations, stimulus control, sleep restriction, relaxation techniques, and cognitive restructuring targeting maladaptive sleep-related beliefs [5]. Despite its strong empirical support, access to face-to-face delivery is often associated with long waiting lists, limited availability of trained providers, and cost barriers. Pharmacological treatments, including hypnotic and sedative medications, are also commonly used, particularly in the short term; however, concerns regarding side effects, tolerance, dependency, and long-term efficacy limit their suitability as a primary treatment strategy [4]. Together, these limitations highlight the need to better understand the self-initiated strategies that individuals use to facilitate sleep onset or overcome sleep initiation difficulties.

Given that sleep initiation problems are closely tied to presleep cognitive arousal [3], patients frequently use cognitive and attentional strategies to manage racing thoughts at bedtime. These include listening to music, relaxation recordings, white noise, guided imagery, and other forms of auditory stimulation [6-8]. Auditory input may function as a structured attentional anchor, reducing internally generated rumination by redirecting cognitive resources outward. In experimental paradigms that manipulated attentional orientation [9], externally delivered auditory stimulation shifted attentional balance from internal to external sources and was associated with reduced internal distraction, supporting the idea that sound can modulate internally generated cognition.

One increasingly common form of auditory stimulation at bedtime and during the night is audiobook listening. In Sweden, streamed audiobook listening has rapidly grown into one of the most popular formats for literary consumption, especially among middle-aged women. In 2025, 16% of the population reported listening to audiobooks on a regular basis [10], which is a high figure in an international perspective [11]. A large-scale study of audiobook user data in a Swedish context showed that 13.3% of the users had a strong bias toward listening during late evenings and nights [12]—periods that typically coincide with sleep onset or insomnia for most adults—suggesting that this practice is widely adopted as a strategy to facilitate sleep. However, despite its growing popularity, possible associations between bedtime audiobook use and sleep outcomes have received little systematic investigation. There are some studies of audiobook users indicating the link between audiobook listening and bedtime routines [12,13], but they do not address matters related to sleep. Existing evidence is largely limited to clinical or experimental contexts. For example, a quasi-experimental study conducted in an intensive care unit in Turkey [14] found that listening to recorded stories significantly improved subjective sleep quality and reduced pulse and blood pressure compared with standard care. While these findings indicate that structured narrative auditory input may positively influence sleep and physiological arousal under controlled conditions, it remains unclear whether similar associations exist in community samples using audiobooks voluntarily in their natural sleep environments.

Importantly, bedtime audiobook listening may serve different functional roles depending on an individual’s sleep health. For individuals without insomnia, audiobooks may provide comfort, routine, or a soothing background embedded within an already functional sleep system. This interpretation is consistent with broader research on auditory sleep aids, which suggests that external sounds or music may promote relaxation, reduce arousal, and support subjective sleep experiences in some individuals [6-8,14]. For individuals with sleep-onset insomnia, however, audiobooks may function as a cognitive regulation strategy aimed at attenuating presleep worry and racing thoughts. At the same time, insomnia research highlights that strategies used to facilitate sleep can also become strongly associated with sleep initiation and may contribute to perceived reliance on external cues, particularly when individuals feel unable to sleep without them [4]. Whether audiobook use is associated with perceived improvements in sleep—and whether such associations differ according to insomnia severity—has not yet been systematically examined.

This Swedish study sought to address this gap using a cross-sectional, anonymous online survey of 1136 individuals who reported listening to audiobooks at bedtime. Specifically, the study aimed to (1) examine whether the frequency of bedtime audiobook use is associated with perceived helpfulness for falling asleep and overall sleep quality, (2) investigate whether audiobook use is associated with perceived difficulty falling asleep in the absence of an audiobook, (3) explore whether listening motives differ according to sleep-onset insomnia status, and (4) assess whether insomnia severity moderates the associations between audiobook use and perceived sleep-related outcomes.

## Methods

### Study Design and Participants

This study was based on data from a cross-sectional online survey conducted in Sweden to investigate audiobook listening habits, with particular emphasis on bedtime use and its perceived association with sleep. The survey was administered in Swedish using the REDCap platform and consisted of 32 items covering bedtime audiobook use, listening frequency, genre preferences, stability of audiobook choice, primary motives for listening, perceived effects on sleep onset and overall sleep quality, and symptoms of sleep-onset insomnia.

Data collection took place between January 23, 2026, and February 25, 2026 (34 d). The survey was disseminated through multiple recruitment channels, including social media platforms (of particular importance is the Swedish Facebook group “*Vi som älskar ljudböcker*” [“We who love audiobooks”], a public group dedicated to discussing audiobooks and holding >86,000 members), flyers posted on the campus of Uppsala University, and coverage by regional media outlets in Uppsala. National dissemination included an interview on a Swedish public radio program about culture (Swedish Radio P1 “Kulturnytt,” February 2, 2026), an article in *Svensk Bokhandel* (February 2, 2026), and the *Boktugg* newsletter (February 6, 2026), distributed under the heading “*Ljudbokssömn*.” All recruitment channels primarily targeted book-oriented audiences.

Participants were anonymous volunteers residing in Sweden. Individuals were instructed not to participate if they were aged <18 years. Participation was voluntary and uncompensated. To enhance data quality and reduce the risk of automated or inattentive responses, an attention-check item was included at the beginning of the survey. This item consisted of a simple arithmetic question (4+1) presented in a free-response format. Participants were also asked whether they had previously completed the survey; responses indicating prior participation were excluded.

Participants were excluded if they did not use audiobooks at bedtime, failed the attention-check item, indicated prior participation, or discontinued the survey before reaching sections containing the exposure, outcome, and covariate variables required for the present analyses. Following these exclusions, no missing data remained in the variables included in the regression models.

### Survey Development and Measures

The survey was developed by the authors for the purposes of this study. Sleep-related items were informed by established instruments commonly used to assess insomnia symptoms and sleep quality, including the Insomnia Severity Index [15] and the Pittsburgh Sleep Quality Index [16]. Audiobook-related items were informed by previous research on audiobook use conducted by KB [10,12], whose work has examined audiobook listening practices and motivations. The questionnaire was designed to capture audiobook listening behaviors, perceived sleep-related effects, and symptoms of sleep-onset insomnia within a single online survey. Survey items were presented in a fixed order and were not randomized. The survey was not formally pilot tested prior to data collection. As the listening-motive items were designed to represent distinct reasons for bedtime audiobook use rather than indicators of a single underlying construct, internal consistency analyses were not performed.

As summarized in Table 1, survey items were organized into demographic, audiobook-related, and sleep-related domains. Demographic variables included age group, gender identity, living alone (derived from household size), and sleeping alone, as these factors may influence sleep patterns and bedtime routines [17-19]. Audiobook-related variables comprised the frequency of bedtime audiobook use (less often, several times per week, and every day), genre preference (fiction vs nonfiction or other genres), stability of audiobook choice (usually the same audiobook vs varying audiobooks), and the primary reason for bedtime listening (to fall asleep, reduce worry or racing thoughts, lullaby or comforting auditory background, mask environmental noise, or other reasons). For analyses of listening motives, separate binary indicators were created for each primary motive category.

**Table 1.** Summary of survey domains and variables included in the present analyses.

Domain	Variables
Demographic variables	Age group, gender identity, living alone (derived from household size), and sleeping alone
Audiobook-related variables	Frequency of bedtime audiobook use, bedtime genre preference, stability of audiobook choice, and primary reason for bedtime listening
Perceived sleep-related outcomes	Helpfulness of audiobooks for falling asleep, perceived impact on overall sleep, and difficulty falling asleep without an audiobook
Sleep characteristics	Habitual sleep onset latency, frequency of difficulty initiating sleep, and duration of sleep initiation difficulties
Derived sleep variable	Sleep-onset insomnia symptom status (absent, subthreshold, and clinical level), derived from frequency and duration of sleep initiation difficulties

Perceived sleep-related outcomes included self-reported helpfulness of audiobooks for falling asleep (somewhat or very helpful vs do not help or worsen), perceived impact on overall sleep (helpful vs not helpful), and perceived difficulty falling asleep without an audiobook (somewhat or much harder vs not harder or better). These outcomes were dichotomized for regression analyses. Sleep-related variables included habitual sleep-onset latency (<30 vs ≥30 min), frequency of difficulty initiating sleep (never, less often, 1‐2 d/wk, or ≥3 d/wk), and duration of sleep difficulties (sporadically, <3 mo, or ≥3 mo). Clinical-level sleep-onset insomnia status was derived by combining reported frequency and duration of sleep initiation difficulties in accordance with symptom-based insomnia criteria [20]. Clinical-level sleep-onset insomnia was defined as difficulty falling asleep at least three times per week, persisting for 3 months or longer. Subthreshold sleep-onset insomnia was defined as either difficulty falling asleep 1 to 2 times per week for 3 months or longer or difficulty falling asleep 3 or more times per week but for less than 3 months.

Additional survey items assessed literary aspects of audiobook use, including narrator voice characteristics and specific fiction subtypes; however, these variables were not included in the present analyses, as the primary objective was to examine associations between bedtime audiobook listening patterns and sleep-related outcomes. Analyses of these literary aspects are planned for a separate manuscript.

### Statistical Analysis

All outcomes were analyzed using modified Poisson regression models with robust variance estimation to estimate prevalence ratios (PRs). Binary outcomes included perceived helpfulness for falling asleep, perceived impact on overall sleep, difficulty falling asleep without an audiobook, genre preference (fiction vs nonfiction), stability of audiobook choice (usually the same vs varying), and primary listening motives. The perceived sleep-related outcomes (helpfulness for falling asleep, impact on overall sleep, and difficulty falling asleep without an audiobook), although originally measured on ordinal scales, were dichotomized prior to analysis to facilitate straightforward interpretation of the results and because the primary aim was to distinguish participants reporting a positive or meaningful sleep-related effect from those who did not. For primary listening motives, separate modified Poisson regression models were conducted for each motive category. For all dichotomous outcomes, variables were coded such that the presence of the outcome of interest was assigned a value of 1 and the reference category a value of 0. Accordingly, PRs >1 indicate a higher prevalence of the coded outcome (value=1), whereas PRs <1 indicate a lower prevalence relative to the reference group.

Reference categories for predictors were defined as the lowest exposure level (“less often” for audiobook frequency) and the absence of sleep-onset insomnia symptoms. Adjusted models were controlled for age group (4 levels), gender identity (3 levels), living alone (binary), and sleeping alone (binary). These variables were selected a priori as potential confounders because they were considered likely to be associated with both audiobook listening practices [10,12,13] and sleep-related outcomes [17-19,21,22]. In addition, crude (unadjusted) models, including only audiobook frequency and sleep-onset insomnia symptom status, were estimated for comparison with the adjusted analyses.

As a post hoc sensitivity analysis, all regression models were repeated in the female-only subsample (n=1027). This analysis was conducted because women represented the vast majority of the sample (>90%), and the aim was to examine whether the observed associations were consistent within this more homogeneous subgroup.

For key models, interaction terms between audiobook listening frequency and sleep-onset insomnia symptom status were included to assess whether associations differed by symptom severity. aPRs with 95% CIs are reported. Wald statistics were derived from the adjusted modified Poisson regression models. For descriptive prevalence estimates of the primary exposures and outcomes, Wilson 95% CIs were calculated. Overall, statistical significance was defined as *P*<.05 (2-tailed).

### Ethical Considerations

The study procedures adhered to the Declaration of Helsinki and were approved by the Regional Ethical Review Board in Uppsala, Sweden (Dnr 2025-01074-01). Before accessing the survey, participants were presented with information describing the purpose of the study, namely, to investigate audiobook listening and sleep. The information page also stated that individuals aged <18 years should not participate. Participants were required to indicate electronically that they had read and understood the study information and agreed to participate before accessing the survey questions. Participation was anonymous. No personally identifiable information, contact information, IP addresses, or participant codes were collected or retained. Survey responses were recorded anonymously using the REDCap platform and were stored and analyzed in an anonymized form. Participation was voluntary, and no financial compensation or other incentives were provided.

## Results

### Cohort Characteristics

A total of 1641 responses were received. After applying the predefined exclusion criteria, 1136 (69%) participants were retained for analysis. Exclusions comprised respondents who did not use audiobooks at bedtime (n=226, 14%), failed the attention-check item (n=148, 9%), discontinued the survey before reaching sections containing the exposure, outcome, and covariate variables required for the present analyses (n=93, 6%), or indicated prior participation (n=38, 2%).

Following these exclusions, the final analytical sample comprised 1136 participants (aged ≥50 years: n=600, 53%; women: n=1027, 90%), with most living with others (n=877, 77%) and 41% (n=469) sleeping alone. Bedtime audiobook listening was common: 53% (n=601) reported daily use (95% CI 50.0‐55.8), 30% (n=338) several times per week (95% CI 27.1‐32.4), and 17% (n=197) less often (95% CI 15.1‐19.5). Most participants preferred fiction (n=824, 73%, 95% CI 69.9‐75.1) and usually listened to the same audiobook at bedtime (n=792, 70%, 95% CI 67.1‐72.4). The primary motivations for bedtime audiobook use were reducing worry or racing thoughts (n=538, 47%, 95% CI 44.5‐50.3) and helping to fall asleep (n=348, 31%, 95% CI 28.0‐33.3).

Overall, 89% (n=1011) found audiobooks somewhat or very helpful for falling asleep (95% CI 87.0‐90.7), and 83% (n=940) reported that falling asleep without an audiobook was somewhat or much harder (95% CI 80.6‐84.9). Sleep-onset insomnia symptoms were reported in 38% (n=427) of the cohort (95% CI 34.8‐40.4), including 19% (n=214) with subthreshold sleep-onset insomnia symptoms (95% CI 16.6‐21.1) and 19% (n=213) with clinical-level sleep-onset insomnia symptoms (95% CI 16.5‐21.0). These estimates reflect the self-selected study sample and should not be interpreted as population prevalence estimates. Additional cohort characteristics stratified by bedtime audiobook listening frequency and sleep-onset insomnia symptom status are presented in Tables 2 and 3, respectively.

**Table 2.** Survey cohort characteristics by audiobook frequency.

Variable	Total cohort	Frequency of bedtime audiobook use		
		Less often	Several times per week	Every day
Participants, n (%)	1136 (100)	197 (17.3)	338 (29.8)	601 (52.9)
Gender identity, n (%)				
Female	1027 (90.4)	182 (92.4)	303 (89.6)	542 (90.2)
Male	104 (9.2)	14 (7.1)	35 (10.4)	55 (9.2)
Unspecified	5 (0.4)	1 (0.5)	0 (0)	4 (0.7)
Age range (y), n (%)				
18‐29	87 (7.7)	28 (14.2)	27 (8.0)	32 (5.3)
30‐49	449 (39.5)	73 (37.1)	140 (41.4)	236 (39.3)
50‐64	439 (38.6)	69 (35.0)	130 (38.5)	240 (39.9)
≥65	161 (14.2)	27 (13.7)	41 (12.1)	93 (15.5)
Living alone, n (%)				
Yes	259 (22.8)	34 (17.3)	78 (23.1)	147 (24.5)
No	877 (77.2)	163 (82.7)	260 (76.9)	454 (75.5)
Sleeping alone, n (%)				
Yes	469 (41.3)	66 (33.5)	137 (40.5)	266 (44.3)
No	667 (58.7)	131 (66.5)	201 (59.5)	335 (55.7)
Bedtime audiobook genre, n (%)				
Fiction	824 (72.5)	121 (61.4)	241 (71.3)	462 (76.9)
Nonfiction or other genres	312 (27.5)	76 (38.6)	97 (28.7)	139 (23.1)
Same audiobook at bedtime, n (%)				
Usually the same	792 (69.7)	127 (64.5)	224 (66.3)	441 (73.4)
Varies	344 (30.3)	70 (35.5)	114 (33.7)	160 (26.6)
Primary reason for bedtime listening, n (%)				
To fall asleep	348 (30.6)	49 (24.9)	102 (30.2)	197 (32.8)
Reduce worry or racing thoughts	538 (47.4)	102 (51.8)	161 (47.6)	275 (45.8)
Lullaby or comforting auditory background	190 (16.7)	26 (13.2)	57 (16.9)	107 (17.8)
Mask environmental noise	21 (1.8)	3 (1.5)	7 (2.1)	11 (1.8)
Other or none of the above	39 (3.4)	17 (8.6)	11 (3.3)	11 (1.8)
Audiobook helps for falling asleep, n (%)				
Somewhat or very helpful	1011 (89.0)	143 (72.6)	299 (88.5)	569 (94.7)
Do not help or worsen	125 (11.0)	54 (27.4)	39 (11.5)	32 (5.3)
Audiobook impact on overall sleep, n (%)				
Helpful	599 (52.7)	68 (34.5)	166 (49.1)	365 (60.7)
Not helpful	537 (47.3)	129 (65.5)	172 (50.9)	236 (39.3)
Difficulty falling asleep without audiobook, n (%)				
Somewhat or much harder	940 (82.7)	97 (49.2)	277 (82.0)	566 (94.2)
Not harder or better	196 (17.3)	100 (50.8)	61 (18.0)	35 (5.8)
Difficulty falling asleep—frequency, n (%)				
Never	132 (11.6)	26 (13.2)	34 (10.1)	72 (12.0)
Less often	498 (43.8)	83 (42.1)	137 (40.5)	278 (46.3)
1‐2 days per week	288 (25.4)	56 (28.4)	106 (31.4)	126 (21.0)
≥3 days per week	218 (19.2)	32 (16.2)	61 (18.0)	125 (20.8)
Difficulty falling asleep—duration, n (%)				
Not applicable (answered “never” to frequency question)	132 (11.6)	26 (13.2)	34 (10.1)	72 (12.0)
Sporadically	355 (31.3)	68 (34.5)	116 (34.3)	171 (28.5)
Last 3 months	43 (3.8)	7 (3.6)	13 (3.8)	23 (3.8)
Since several years	606 (53.3)	96 (48.7)	175 (51.8)	335 (55.7)
Sleep-onset insomnia, n (%)				
No	709 (62.4)	128 (65.0)	204 (60.4)	377 (62.7)
Subthreshold	214 (18.8)	37 (18.8)	75 (22.2)	102 (17.0)
Clinical	213 (18.8)	32 (16.2)	59 (17.5)	122 (20.3)

**Table 3.** Survey cohort characteristics by sleep-onset insomnia symptom status.

Variable	Total cohort	Sleep-onset insomnia		
		Absent	Subthreshold	Clinical
Participants, n (%)	1136 (100)	709 (62.4)	214 (18.8)	213 (18.8)
Gender identity, n (%)				
Female	1027 (90.4)	643 (90.7)	191 (89.3)	193 (90.6)
Male	104 (9.2)	63 (8.9)	22 (10.3)	19 (8.9)
Unspecified	5 (0.4)	3 (0.4)	1 (0.5)	1 (0.5)
Age range (y), n (%)				
18‐29	87 (7.7)	50 (7.1)	18 (8.4)	19 (8.9)
30‐49	449 (39.5)	302 (42.6)	79 (36.9)	68 (31.9)
50‐64	439 (38.6)	266 (37.5)	87 (40.7)	86 (40.4)
≥65	161 (14.2)	91 (12.8)	30 (14.0)	40 (18.8)
Living alone, n (%)				
Yes	259 (22.8)	163 (23.0)	47 (22.0)	49 (23.0)
No	877 (77.2)	546 (77.0)	167 (78.0)	164 (77.0)
Sleeping alone, n (%)				
Yes	469 (41.3)	281 (39.6)	84 (39.3)	104 (48.8)
No	667 (58.7)	428 (60.4)	130 (60.7)	109 (51.2)
Frequency of bedtime audiobook use, n (%)				
Less often	197 (17.3)	128 (18.1)	37 (17.3)	32 (15.0)
Several times per week	338 (29.8)	204 (28.8)	75 (35.0)	59 (27.7)
Every day	601 (52.9)	377 (53.2)	102 (47.7)	122 (57.3)
Bedtime audiobook genre, n (%)				
Fiction	824 (72.5)	519 (73.2)	164 (76.6)	141 (66.2)
Nonfiction or other genres	312 (27.5)	190 (26.8)	50 (23.4)	72 (33.8)
Same audiobook at bedtime, n (%)				
Usually the same	792 (69.7)	501 (70.7)	153 (71.5)	138 (64.8)
Varies	344 (30.3)	208 (29.3)	61 (28.5)	75 (35.2)
Primary reason for bedtime listening, n (%)				
To fall asleep	348 (30.6)	210 (29.6)	61 (28.5)	77 (36.2)
Reduce worry or racing thoughts	538 (47.4)	303 (42.7)	122 (57.0)	113 (53.1)
Lullaby or comforting auditory background	190 (16.7)	153 (21.6)	23 (10.7)	14 (6.6)
Mask environmental noise	21 (1.8)	11 (1.6)	6 (2.8)	4 (1.9)
Other or none of the above	39 (3.4)	32 (4.5)	2 (0.9)	5 (2.3)
Audiobook helps for falling asleep, n (%)				
Somewhat or very helpful	1011 (89.0)	642 (90.6)	189 (88.3)	180 (84.5)
Do not help or worsen	125 (11.0)	67 (9.4)	25 (11.7)	33 (15.5)
Audiobook impact on overall sleep, n (%)				
Helpful	599 (52.7)	348 (49.1)	99 (46.3)	90 (42.3)
Not helpful	537 (47.3)	361 (50.9)	115 (53.7)	123 (57.7)
Difficulty falling asleep without audiobook, n (%)				
Somewhat or much harder	940 (82.7)	565 (79.7)	187 (87.4)	188 (88.3)
Not harder or better	196 (17.3)	144 (20.3)	27 (12.6)	25 (11.7)
Difficulty falling asleep—frequency, n (%)				
Never	132 (11.6)	132 (18.6)	0 (0)	0 (0)
Less often	498 (43.8)	498 (70.2)	0 (0)	0 (0)
1‐2 days per week	288 (25.4)	79 (11.1)	209 (97.7)	0 (0)
≥3 days per week	218 (19.2)	0 (0)	5 (2.3)	213 (100)
Difficulty falling asleep—duration, n (%)				
Not applicable (answered “never” to frequency question)	132 (11.6)	132 (18.6)	0 (0)	0 (0)
Sporadically	355 (31.3)	350 (49.4)	5 (2.3)	0 (0)
Last 3 months	43 (3.8)	21 (3.0)	12 (5.6)	10 (4.7)
Since several years	606 (53.3)	206 (29.1)	197 (92.1)	203 (95.3)

### Associations Between Bedtime Audiobook Listening and Perceived Sleep-Related Outcomes

In the adjusted analyses, compared with occasional bedtime audiobook listening, listening several times per week and daily were both associated with higher perceived helpfulness for falling asleep (aPR 1.23, 95% CI 1.12‐1.35 and aPR 1.32, 95% CI 1.21‐1.44, respectively), greater perceived overall sleep benefit (aPR 1.41, 95% CI 1.13‐1.75 and aPR 1.73, 95% CI 1.41‐2.12, respectively), and greater perceived difficulty falling asleep without an audiobook (aPR 1.67, 95% CI 1.44‐1.94 and aPR 1.93, 95% CI 1.67‐2.22, respectively). Crude PRs and aPRs for audiobook listening frequency are presented in Table 4.

**Table 4.** Associations between bedtime audiobook listening frequency and study outcomes^a^.

Outcome	Audiobook at bedtime frequency			
	Several times per week vs less often		Daily vs less often	
	cPRs^b^ (95% CI)	aPRs^c^ (95% CI)	cPRs (95% CI)	aPRs (95% CI)
Sleep-related outcomes				
Helpful for falling asleep	1.22 (1.11‐1.34)	1.23 (1.12‐1.35)	1.30 (1.20‐1.42)	1.32 (1.21‐1.44)
Overall sleep benefit	1.42 (1.14‐1.77)	1.41 (1.13‐1.75)	1.76 (1.44‐2.16)	1.73 (1.41‐2.12)
Difficulty falling asleep without audiobook	1.66 (1.43‐1.93)	1.67 (1.44‐1.94)	1.91 (1.66‐2.21)	1.93 (1.67‐2.22)
Primary motives for bedtime audiobook listening				
To fall asleep	1.21 (0.91‐1.62)	1.20 (0.90‐1.61)	1.32 (1.01‐1.72)	1.27 (0.97‐1.67)
Reducing worry or racing thoughts	0.92 (0.77‐1.10)	0.92 (0.77‐1.09)	0.88 (0.75‐1.04)	0.90 (0.77‐1.06)
Using audiobooks as a lullaby or comforting auditory background	1.28 (0.83‐1.96)	1.30 (0.85‐1.98)	1.35 (0.91‐2.01)	1.35 (0.91‐2.01)
Audiobook preferences and listening patterns				
Nonfiction	0.74 (0.58‐0.95)	0.76 (0.60‐0.96)	0.60 (0.48‐0.75)	0.61 (0.49‐0.77)
Same audiobook at bedtime	1.03 (0.90‐1.17)	1.02 (0.89‐1.16)	1.14 (1.02‐1.28)	1.13 (1.00‐1.26)

a Values estimated using modified Poisson regression with robust variance. Adjusted models included age group, gender identity, living alone, sleeping alone, and sleep-onset insomnia symptom status. The reference category was occasional bedtime audiobook listening.

b cPR: crude prevalence ratio.

c aPR: adjusted prevalence ratio.

In the adjusted analyses, participants with clinical-level sleep-onset insomnia symptoms were less likely to report that audiobooks were helpful for falling asleep than those without sleep-onset insomnia symptoms (aPR 0.93, 95% CI 0.87‐0.98). Participants with subthreshold and clinical-level sleep-onset insomnia symptoms were more likely to report difficulty falling asleep without an audiobook (subthreshold: aPR 1.10, 95% CI 1.04‐1.17; and clinical level: aPR 1.09, 95% CI 1.03‐1.15). aPRs for perceived overall sleep benefit were close to the null (subthreshold: aPR 1.06, 95% CI 0.92‐1.23; and clinical level: aPR 1.09, 95% CI 0.96‐1.25). Crude PRs and aPRs for sleep-onset insomnia symptom status are presented in Table 5. No significant interactions were observed between audiobook listening frequency and sleep-onset insomnia symptom status for any sleep-related outcome (all *P*>.05).

**Table 5.** Associations between sleep-onset insomnia symptom status and study outcomes^a^.

Outcome	Sleep-onset insomnia symptom status			
	Subthreshold vs absent		Clinical level vs absent	
	cPRs^b^ (95% CI)	aPRs^c^ (95% CI)	cPRs (95% CI)	aPRs (95% CI)
Sleep-related outcomes				
Helpful for falling asleep	0.98 (0.92‐1.03)	0.98 (0.93‐1.03)	0.93 (0.88‐0.99)	0.93 (0.87‐0.98)
Overall sleep benefit	1.06 (0.91‐1.22)	1.06 (0.92‐1.23)	1.13 (0.99‐1.30)	1.09 (0.96‐1.15)
Difficulty falling asleep without audiobook	1.10 (1.03‐1.17)	1.10 (1.04‐1.17)	1.11 (1.04‐1.18)	1.09 (1.03‐1.15)
Primary motives for bedtime audiobook listening				
To fall asleep	0.96 (0.76‐1.22)	0.96 (0.75‐1.22)	1.22 (0.99‐1.51)	1.18 (0.96‐1.46)
Reducing worry or racing thoughts	1.33 (1.16‐1.54)	1.34 (1.16‐1.55)	1.24 (1.07‐1.45)	1.28 (1.10‐1.49)
Using audiobooks as a lullaby or comforting auditory background	0.50 (0.33‐0.75)	0.50 (0.33‐0.75)	0.31 (0.18‐0.52)	0.29 (0.17‐0.50)
Audiobook preferences and listening patterns				
Nonfiction	0.87 (0.67‐1.14)	0.86 (0.67‐1.12)	1.26 (1.01‐1.58)	1.30 (1.04‐1.61)
Same audiobook at bedtime	1.01 (0.92‐1.12)	1.02 (0.92‐1.12)	0.92 (0.82‐1.02)	0.91 (0.82‐1.02)

a Values estimated using modified Poisson regression with robust variance. Adjusted models included age group, gender identity, living alone, sleeping alone, and audiobook listening frequency. The reference category was absence of sleep-onset insomnia symptoms.

b cPR: crude prevalence ratio.

c aPR: adjusted prevalence ratio.

### Primary Motives for Bedtime Audiobook Listening

As relatively few participants selected “mask environmental noise” (n=21, 1.8%) or “other or unspecified” (n=39, 3.4%) as their primary listening motive, these categories were not emphasized in the presentation of the results. aPRs for audiobook listening frequency were close to the null for reporting listening primarily to fall asleep, reducing worry or racing thoughts, or using audiobooks as a lullaby or comforting auditory background (Table 4).

Compared with participants without sleep-onset insomnia symptoms, those with subthreshold (aPR 1.34, 95% CI 1.16‐1.55) and clinical-level (aPR 1.28, 95% CI 1.10‐1.49) symptoms were more likely to report listening to audiobooks primarily to reduce worry or racing thoughts. Conversely, participants with subthreshold (aPR 0.50, 95% CI 0.33‐0.75) and clinical-level (aPR 0.29, 95% CI 0.17‐0.50) symptoms were less likely to report listening primarily for lullaby-like or comforting auditory background purposes. aPRs for endorsing “to fall asleep” as the primary listening motive were close to the null (Table 5). No significant interactions were observed between audiobook listening frequency and sleep-onset insomnia symptom status for any motive category (all *P*>.05).

### Audiobook Preferences and Listening Patterns

Compared with occasional listeners, participants listening several times per week (aPR 0.76, 95% CI 0.60‐0.96) and daily (aPR 0.61, 95% CI 0.49‐0.77) were less likely to prefer nonfiction, indicating a greater preference for fiction. Daily listeners were also slightly more likely to report usually listening to the same audiobook at bedtime (aPR 1.13, 95% CI 1.00‐1.26), whereas the estimate for several-times-per-week listeners was close to the null (aPR 1.02, 95% CI 0.89‐1.16; Table 4).

Participants with clinical-level sleep-onset insomnia symptoms were more likely to prefer nonfiction than those without sleep-onset insomnia symptoms (aPR 1.30, 95% CI 1.04‐1.61), whereas aPRs for the stability of audiobook choice were close to the null for both subthreshold (aPR 1.02, 95% CI 0.92‐1.12) and clinical-level symptoms (aPR 0.91, 95% CI 0.82‐1.02; Table 5). No significant interactions were observed (all *P*>.05).

### Reanalysis Among Female Subsample

As presented in Tables 6 and 7, the main findings from the full cohort were consistent among women, who accounted for more than 90% of respondents. Specifically, adjusted PRs showed similar magnitude and direction across sleep-related outcomes, primary motives for audiobook use, and audiobook listening preferences. The associations between audiobook listening frequency and perceived sleep-related outcomes were nearly unchanged in the female-only subsample, as were the associations between sleep-onset insomnia symptom status and difficulty falling asleep without an audiobook. Overall, these findings suggest that the primary results were robust and were not substantially influenced by the inclusion of the smaller proportion of male and nonbinary participants in the full sample.

**Table 6.** Associations between bedtime audiobook listening frequency and study outcomes in the female subsample (n=1027)^a^.

Outcome	Audiobook at bedtime frequency			
	Several times per week vs less often		Daily vs less often	
	cPRs^b^ (95% CI)	aPRs^c^ (95% CI)	cPRs (95% CI)	aPRs (95% CI)
Sleep-related outcomes				
Helpful for falling asleep	1.20 (1.09‐1.32)	1.21 (1.10‐1.33)	1.29 (1.18‐1.41)	1.30 (1.19‐1.42)
Overall sleep benefit	1.37 (1.09‐1.73)	1.36 (1.08‐1.72)	1.76 (1.43‐2.18)	1.74 (1.40‐2.15)
Difficulty falling asleep without audiobook	1.72 (1.46‐2.02)	1.72 (1.46‐2.02)	1.97 (1.69‐2.30)	1.98 (1.70‐2.31)
Primary motives for bedtime audiobook listening				
To fall asleep	1.19 (0.88‐1.61)	1.18 (0.87‐1.59)	1.31 (0.99‐1.72)	1.26 (0.95‐1.67)
Reducing worry or racing thoughts	0.94 (0.78‐1.13)	0.94 (0.78‐1.13)	0.90 (0.76‐1.07)	0.92 (0.78‐1.09)
Using audiobooks as a lullaby or comforting auditory background	1.18 (0.76‐1.82)	1.21 (0.78‐1.85)	1.23 (0.82‐1.83)	1.23 (0.83‐1.82)
Audiobook preferences and listening patterns				
Nonfiction	0.73 (0.56‐0.95)	0.74 (0.57‐0.97)	0.60 (0.47‐0.77)	0.62 (0.48‐0.79)
Same audiobook at bedtime	0.99 (0.87‐1.13)	0.98 (0.86‐1.13)	1.14 (1.01‐1.28)	1.13 (1.00‐1.27)

a Values estimated using modified Poisson regression with robust variance. Adjusted models included age group, living alone, sleeping alone, and sleep-onset insomnia symptom status. The reference category was occasional bedtime audiobook listening.

b cPR: crude prevalence ratio.

c aPR: adjusted prevalence ratio.

**Table 7.** Associations between sleep-onset insomnia symptom status and study outcomes in the female subsample (n=1027)^a^.

Outcome	Sleep-onset insomnia symptom status			
	Subthreshold vs absent		Clinical level vs absent	
	cPRs^b^ (95% CI)	aPRs^c^ (95% CI)	cPRs (95% CI)	aPRs (95% CI)
Sleep-related outcomes				
Helpful for falling asleep	0.98 (0.93‐1.04)	0.98 (0.93‐1.04)	0.95 (0.89‐1.01)	0.94 (0.89‐1.00)
Overall sleep benefit	1.07 (0.92‐1.25)	1.08 (0.93‐1.26)	1.16 (1.00‐1.33)	1.12 (0.97‐1.29)
Difficulty falling asleep without audiobook	1.10 (1.02‐1.17)	1.10 (1.03‐1.17)	1.11 (1.04‐1.19)	1.09 (1.03‐1.16)
Primary motives for bedtime audiobook listening				
To fall asleep	1.04 (0.81‐1.33)	1.03 (0.81‐1.32)	1.24 (0.99‐1.55)	1.19 (0.96‐1.49)
Reducing worry or racing thoughts	1.27 (1.08‐1.49)	1.28 (1.09‐1.50)	1.23 (1.05‐1.45)	1.26 (1.08‐1.48)
Using audiobooks as a lullaby or comforting auditory background	0.51 (0.33‐0.78)	0.51 (0.33‐0.79)	0.29 (0.16‐0.51)	0.28 (0.16‐0.50)
Audiobook preferences and listening patterns				
Nonfiction	0.84 (0.62‐1.14)	0.84 (0.62‐1.15)	1.33 (1.05‐1.70)	1.37 (1.08‐1.74)
Same audiobook at bedtime	1.02 (0.92‐1.13)	1.03 (0.93‐1.14)	0.92 (0.82‐1.03)	0.91 (0.81‐1.03)

a Values are estimated using modified Poisson regression with robust variance. Adjusted models included age group, living alone, sleeping alone, and audiobook listening frequency. The reference category was absence of sleep-onset insomnia symptoms.

b cPR: crude prevalence ratio.

c aPR: adjusted prevalence ratio.

## Discussion

### Principal Findings

This cross-sectional survey of 1136 adults residing in Sweden who listen to audiobooks at bedtime suggests that bedtime audiobook use may be particularly common among individuals with sleep initiation difficulties, with 38% reporting subthreshold or clinical-level sleep-onset insomnia. Although the recruitment strategy precludes general population prevalence estimates, the relatively high proportion of participants reporting sleep-onset insomnia is consistent with the possibility that audiobooks are frequently adopted as a self-initiated coping strategy in the context of difficulty falling asleep.

Across outcomes, listening frequency was consistently associated with perceived sleep-related outcomes. Participants who listened daily or several times per week had a higher adjusted prevalence of reporting that audiobooks helped them fall asleep and improved overall sleep. At the same time, frequent listeners were markedly more likely to report difficulty falling asleep when an audiobook was unavailable. These associations did not differ by insomnia status, suggesting that habitual use was associated with both perceived benefit and perceived reliance across levels of sleep health. However, because of the cross-sectional design, the direction of this association cannot be determined. Frequent audiobook use may contribute to perceived reliance, but it is equally plausible that individuals who already experience greater difficulty falling asleep are more likely to adopt audiobooks as a coping strategy and consequently use them more frequently. Supporting this interpretation, frequent listening was also associated with a greater likelihood of repeatedly choosing the same audiobook. Repeated selection of the same audiobook may reflect the development of a stable bedtime listening routine and could represent a consistent sleep-associated cue. Importantly, it is not only repeating the same material per se but also repeated exposure to content that carries personal meaning or emotional significance—such as narratives that evoke comforting memories or emotional engagement—that may enhance relaxation and the sense of safety at bedtime. Within stimulus control and conditioning frameworks, repeated pairing of a stable, meaningful environmental cue with sleep onset can strengthen associative links over time [23]. Bedtime audiobooks may therefore become integrated into learned sleep routines, particularly when exposure is regular and contextually restricted (eg, confined to bedtime and limited in duration via timer functions). However, the same pattern may also be interpreted as reflecting conditioned reliance on an external cue for sleep initiation. The markedly higher aPRs for reporting difficulty falling asleep without an audiobook among daily listeners are compatible with either interpretation. From a conditioning perspective, repeated pairing of audiobook exposure with sleep onset could strengthen a beneficial sleep-associated routine, but it could also increase perceived dependence on the presence of the audiobook when attempting to fall asleep. The present data cannot distinguish between these possibilities.

The relationship between bedtime audiobook listening and stimulus control principles in CBT-I [24] is also worth considering. On the one hand, if audiobook use is restricted to the sleep period, limited in duration through timer functions, and consistently paired with sleep onset, it could potentially function as a stable sleep-associated cue. On the other hand, stimulus control interventions traditionally aim to strengthen the association between the bed and sleep while minimizing reliance on external aids or activities to initiate sleep [4,24]. From this perspective, frequent audiobook use could be viewed as creating dependence on an external cue rather than strengthening sleep itself as the primary conditioned response. The findings of this study cannot distinguish between these possibilities. Indeed, the observed association between frequent audiobook use and greater perceived difficulty falling asleep without an audiobook is compatible with either interpretation. Determining whether bedtime audiobooks complement, compete with, or modify stimulus control processes will require longitudinal and experimental studies, particularly within clinical populations receiving CBT-I.

Importantly, the findings of this study should be interpreted alongside the limited experimental literature on audiobooks and sleep. Existing studies have provided some evidence that bedtime audio-based interventions may influence subjective sleep experiences [14], but findings specifically for audiobooks remain limited and do not establish consistent effects across sleep outcomes. For example, a prior bedtime intervention study found improvements in sleep quality among participants assigned to music, whereas the audiobook condition did not show comparable improvement [25]. These differences may reflect variation in intervention content, study populations, outcome measures, and whether effects were assessed through subjective perceptions or objective sleep parameters. This study extends this literature by examining how individuals perceive and use audiobooks in relation to sleep, particularly among those experiencing sleep-onset difficulties.

Audiobook-sleep associations appeared to differ as a function of sleep-onset insomnia status, revealing a more nuanced functional pattern. Individuals with clinical-level sleep-onset insomnia symptoms were less likely than those without insomnia to report that audiobooks directly helped them fall asleep, yet were more likely to report difficulty falling asleep in the absence of an audiobook. Motive analyses provide context for this apparent paradox. Participants with subthreshold and clinical-level sleep-onset insomnia symptoms were significantly more likely to endorse reducing worry or racing thoughts as their primary reason for listening, whereas those without insomnia were more likely to report listening for calming or lullaby-like purposes. This distinction aligns with the broader literature on the use of sensory and auditory strategies for sleep, where external stimuli may serve different functions, including reducing cognitive arousal, promoting relaxation, or establishing a bedtime routine [6,26,27]. However, this study extends this literature by showing that, in the context of audiobooks specifically, the perceived function of listening may differ according to sleep-related difficulties: individuals with insomnia symptoms appeared more likely to use audiobooks as a cognitive regulation strategy, whereas those without insomnia symptoms more often described their use in terms of comfort or routine. Together, these findings suggest that, among individuals with insomnia symptoms, audiobooks may be perceived primarily as a strategy for managing presleep cognitive activity rather than as a direct sleep-inducing tool.

Cognitive behavioral models of insomnia identify presleep cognitive arousal—particularly worry and racing thoughts—as a central mechanism for maintaining prolonged sleep latency [3,28,29]. Empirical evidence further indicates that heightened presleep cognitive arousal is associated with objective sleep disturbance and reduced sleep efficiency [30]. Within this framework, bedtime audiobooks may provide a structured attentional focus that helps redirect attention away from internally focused thoughts. Consistent with this interpretation, individuals with subthreshold and clinical-level sleep-onset insomnia symptoms were significantly more likely to endorse “reducing worry or racing thoughts” as their primary motive for listening. At the same time, participants with clinical-level sleep-onset insomnia symptoms were less likely to report that audiobooks helped them fall asleep, while simultaneously reporting greater difficulty falling asleep without an audiobook, suggesting a more complex relationship between perceived benefit and reliance on bedtime audiobook use. Moreover, individuals with insomnia were less likely to endorse listening for a soothing or lullaby-like background. Together, these findings suggest that although audiobooks may effectively target cognitive arousal by providing attentional distraction, attenuation of rumination alone may be insufficient to override the broader hyperarousal processes characteristic of clinical insomnia [3,28,29]. This may explain why individuals with clinical-level sleep-onset insomnia symptoms report cognitively oriented motives and greater reliance on audiobooks, yet remain less likely to perceive them as fully resolving sleep initiation difficulties.

Content preferences provided additional nuance. More frequent listeners were more likely to prefer fiction, whereas individuals with clinical-level sleep-onset insomnia symptoms were more likely to select nonfiction. Although these associations were modest and should be interpreted cautiously, they may suggest that different listening preferences are associated with different bedtime listening patterns or functions. Fiction may be more compatible with immersive narrative engagement, whereas nonfiction may reflect a preference for more structured informational content [31]. However, the present data do not allow conclusions regarding the cognitive processes underlying these preferences, and whether different types of audiobook content differentially influence presleep cognitive arousal remains an open empirical question requiring experimental investigation.

Interestingly, endorsement of “to fall asleep” as a primary motive did not differ significantly by insomnia status or listening frequency in the adjusted model. Among individuals without insomnia, this suggests that audiobooks may not be perceived primarily as an active sleep-inducing intervention but rather as part of a comfortable bedtime routine. Among those with insomnia, the lack of strong endorsement of this motive—despite greater reliance and greater endorsement of cognitive regulation motives—further supports the interpretation that audiobooks may mitigate presleep cognitive activity without fully resolving sleep initiation problems. Collectively, the findings point toward distinct functional roles: for individuals with sleep-onset insomnia, audiobooks may operate primarily through attentional redirection away from rumination, whereas for those without insomnia, they may serve more as a stable contextual cue embedded within an already functional sleep system.

An important consideration when interpreting these findings is the potential for reverse causality. The observed association between more frequent bedtime audiobook listening and greater perceived difficulty falling asleep without an audiobook is compatible with multiple explanations that cannot be disentangled in a cross-sectional design. On the one hand, repeated audiobook use could potentially contribute to the development of sleep-related routines or perceived reliance over time. On the other hand, individuals who already experience difficulty falling asleep may be more likely to adopt audiobooks as a coping strategy and subsequently use them more frequently. Similarly, the observed associations between audiobook listening frequency, perceived sleep-related benefits, and sleep-onset insomnia symptom status cannot establish temporal ordering or causality. Consequently, the findings of this study should be interpreted as associations rather than evidence that audiobook use causes either improvements in sleep or increased reliance on audiobooks at bedtime. As the outcomes were based on self-reported perceptions and analyzed as binary variables, the regression estimates should also be interpreted as differences in the likelihood of reporting these experiences rather than as estimates of absolute changes in sleep outcomes. Longitudinal and experimental studies are needed to determine the direction and the mechanisms underlying these relationships.

### Limitations

Although audiobook listening in Sweden is generally most common among middle-aged women [10], the marked female predominance of the sample limits the generalizability of the findings to men and other demographic groups. A sensitivity analysis restricted to women yielded findings that were highly consistent with those observed in the full sample. However, the relatively small number of male participants precluded meaningful gender-stratified analyses, and potential gender differences in bedtime audiobook listening practices and their associations with sleep-related outcomes therefore remain unclear.

Recruitment occurred primarily through audiobook-oriented communities and media channels targeting book-interested audiences, and participation was restricted to individuals who reported using audiobooks at bedtime. Furthermore, the recruitment materials explicitly focused on audiobooks and sleep, which may have preferentially attracted individuals who already perceived audiobooks as affecting their sleep, whether positively or negatively. Consequently, the sample was enriched for audiobook engagement and may have overrepresented individuals with particularly strong views regarding the effects of audiobooks on sleep. This self-selection process may have inflated estimates of perceived sleep benefits and perceived difficulty sleeping without an audiobook, as individuals who viewed audiobooks as having little relevance to their sleep may have been less motivated to participate. It may also help explain the relatively high prevalence of sleep-onset insomnia symptoms observed in the sample. Accordingly, prevalence estimates relating to perceived sleep benefits, perceived reliance on audiobooks, and sleep-onset insomnia symptoms should not be interpreted as representative of either the general population or all audiobook users. Although the direction and magnitude of any resulting bias in the observed associations are difficult to determine, self-selection may have influenced both the exposure and outcome distributions. Replication in independently recruited samples will therefore be important for establishing the generalizability and robustness of the findings.

The study focused exclusively on audiobook listening and did not include a comparator condition, such as bedtime physical book reading. Prior research suggests that physical reading before sleep may confer sleep-related benefits. A randomized pragmatic trial [32] found that participants who read a book in bed before sleep reported improved sleep quality compared with those who did not read before bed, and observational evidence indicates that bedtime reading is associated with reduced sleep onset latency and longer sleep duration. Therefore, the findings of this study should not be interpreted as evidence that audiobooks are superior or inferior to other forms of bedtime reading; rather, they reflect associations within a population of audiobook users.

The study relied on self-reported sleep measures and perceived effects. Objective sleep parameters were not assessed. In addition, although analyses were adjusted for age group, gender, living alone, and sleeping alone, residual confounding from unmeasured factors—including mental health, medication use, chronotype, shift work, and other sleep-related behaviors—cannot be excluded. Interventional studies incorporating polysomnography or actigraphy are necessary to more precisely determine how bedtime audiobook listening influences sleep onset latency, sleep architecture, and sleep continuity across the night.

A relatively large number of statistical tests were conducted across multiple outcomes and subgroup analyses. No formal correction for multiple comparisons was applied, as the analyses were primarily exploratory in nature. Consequently, some statistically significant findings may reflect type I error, and individual associations should be interpreted with appropriate caution. Replication in independent samples is needed to establish the robustness of the observed associations.

This study also did not consider several potentially important characteristics of audiobook exposure or content. Information was not collected regarding listening duration, whether audiobooks were played using a sleep timer, whether listening typically ceased at sleep onset or continued throughout the night, or the extent to which participants remained aware of the audiobook while attempting to fall asleep. In addition, sound-related characteristics such as playback volume, listening device, use of headphones versus speakers, and background audibility were not assessed. The study also did not assess several potentially relevant characteristics of audiobook content, including narrative structure, emotional tone, pacing, cognitive load, or narrator-related factors. Although broad genre preference (fiction vs nonfiction) varied by listening frequency and sleep-onset insomnia symptom status, it remains unclear whether more specific content characteristics differentially influence sleep-related processes. Previous audiobook research further suggests that narrator characteristics play an important role in audiobook selection and listening preferences [33,34]. These factors may influence both the effectiveness of audiobooks as a sleep-related tool and the mechanisms through which audiobook listening interacts with sleep initiation. Future studies should examine these variables in greater detail.

### Conclusions

Notwithstanding these limitations, bedtime audiobooks appear to be perceived as a context-dependent sleep support strategy, with different perceived roles depending on sleep health. Individuals with sleep-onset insomnia symptoms were more likely to report listening to reduce worry or racing thoughts, whereas those without insomnia symptoms more commonly endorsed comfort- or routine-related motives.

Although bedtime audiobooks were widely perceived as helpful—particularly among frequent users—they were not perceived as fully resolving sleep initiation difficulties among individuals with clinical-level sleep-onset insomnia symptoms. Frequent audiobook use was also associated with substantially greater perceived difficulty falling asleep without an audiobook. Owing to the cross-sectional design, the direction of these associations cannot be determined. The findings are consistent both with the possibility that audiobooks become integrated into established bedtime routines over time and with the alternative possibility that individuals experiencing greater difficulty initiating sleep are more likely to adopt and repeatedly use audiobooks as a coping strategy.

Future longitudinal and experimental research should examine whether bedtime audiobooks can be strategically incorporated into behavioral sleep interventions and clarify whether their sleep-related effects reflect attentional distraction, conditioned sleep-associated cues, perceived reliance on external stimuli, or a combination of these processes. Additional work should also investigate whether specific audiobook characteristics—such as listening duration, timer use, narrator characteristics, genre, emotional tone, or cognitive complexity—influence sleep-related outcomes.

## Abbreviations

aPR: adjusted prevalence ratio
CBT-I: cognitive behavioral therapy for insomnia
cPR: crude prevalence ratio
PR: prevalence ratio
